# Symptomatic Extra-Adrenal Myelolipoma in the Spleen

**DOI:** 10.1155/2020/8839178

**Published:** 2020-07-31

**Authors:** Jonathan A. Nitz, Jeremy Huckleby, Elise H. Hwang, Melissa G. Medina, Samuel J. Pera, Sonia T. Orcutt

**Affiliations:** Department of Surgery, University of Illinois College of Medicine at Peoria, Peoria, IL, USA

## Abstract

A 42-year-old male patient presented with intermittent abdominal pain and gastrointestinal discomfort present for 4 years. Work-up included ultrasound and computed tomography, which identified a fat-containing splenic mass 5.6 cm in size. Due to recurrent symptoms, the patient sought medical care again. Subsequent images showed an increase in size to 7.6 cm, which was concerning for neoplasm. This was removed via open splenectomy, which was challenging due to intra-abdominal adhesions despite never having had any abdominal surgery. The patient's recovery was uncomplicated. Pathologic assessment indicated that the mass was a myelolipoma. Extra-adrenal myelolipomas are rare and typically found within the retroperitoneum but are extremely rare within the spleen. This case report adds the 6^th^ such case to the literature and demonstrates the need for it to remain in the differential diagnosis of patients with fatty splenic masses, as well as that splenectomy is an appropriate treatment.

## 1. Introduction

The differential diagnosis for fat-containing splenic masses seen on CT scans range from benign neoplasms to malignancies of mesenchymal origin. Myelolipoma is a rare tumor, but most commonly found in the adrenal gland. It is a rare finding in extra-adrenal locations especially within the spleen [1]. When they contain a large amount of fat, they can be difficult to distinguish radiologically from other fatty neoplasms, including lipoma and liposarcoma. However, they can be differentiated pathologically [1]. We report a case of a patient with myelolipoma of the spleen treated with splenectomy.

## 2. Case Presentation

**Figure 1:**
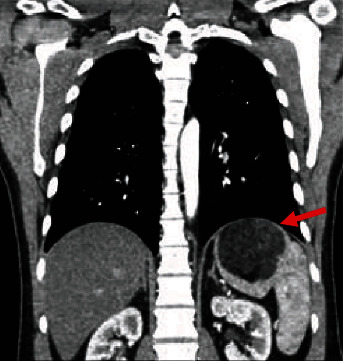
Contrast-enhanced computed tomography coronal image depicting the myelolipoma within the spleen (arrow).

A 42-year-old male patient presented with irregular bowel movements and abdominal pain, requiring multiple hospital admissions. During these admissions, a splenic mass was identified radiographically. While this was deemed not to be the source of the irregular bowel movements, an ultrasound of his abdomen during one of these admissions 3 years prior to our evaluation revealed a left upper quadrant mass. This was subsequently assessed with computed tomography (CT) scan, which identified a fatty mass potentially arising from the spleen or adrenal gland. The mass was initially found to be 5.6 cm in size but was noted to be growing in size on repeat imaging 2 years later, with a subsequent measurement of 6 cm in size. He was advised to see a surgeon, but due to an upcoming move, he delayed further evaluation and work-up. Once settled, he established himself with a primary care physician and underwent CT scan 1 year later showing a 7.6 × 7.1 × 6.4 cm splenic mass (Figure 1), which was larger than before. The mass was largely fat containing with a differential diagnosis including lipoma, myelolipoma, or dermoid cyst. Potential neoplastic etiologies included liposarcoma, which was of primary concern secondary to the growth of the lesion over time. There were no obvious features of de-differentiation or metastasis, and the mass was entirely within the spleen with no involvement of the adrenal gland. On our assessment, the patient was generally well appearing, overweight (BMI 31), with an abdominal exam that was benign with a nonpalpable spleen, and only minimal tenderness to palpation in the left upper quadrant. His heart rate was nontachycardic at 84 beats per minute. His blood pressure was normal at 139/79, and he was afebrile. His laboratory studies included complete blood count (CBC) and complete metabolic panel (CMP). CBC revealed an elevated hemoglobin at 16.8, white blood cell count of 6.6, and platelet count of 200. CMP revealed mild transaminitis with AST and ALT of 42 and 81, respectively, with other values within normal limits.

Percutaneous biopsy was not obtained due to concern for hemorrhage, and that as the mass was growing, surgery was recommended regardless of the biopsy findings. He was counselled on the risks and benefits of open splenectomy, as well as alternatives including observation. As the mass was growing in size and due to concern for sarcoma, the patient wished for operative intervention. An open approach was favored due to the overall size of the spleen along with concern for sarcoma. He gave consent to proceed with the operation. Presplenectomy vaccines were administered 2 weeks preoperatively.

**Figure 2:**
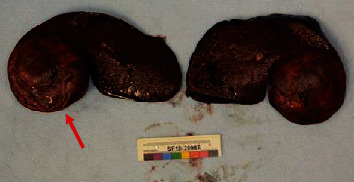
Spleen bisected with intrasplenic myelolipoma (arrow).

Splenectomy was performed through a left subcostal incision. There was significant adhesive disease (presumably inflammatory in nature as he had not had prior abdominal surgeries) in the left upper quadrant, but the spleen was amenable to be removed intact and without violation of the mass. The patient had an unremarkable postoperative course and recovered well. Pathology identified the mass to be 8.5 × 8 × 6 cm, well-circumscribed, and with a yellow/tan heterogeneous cut surface (Figure 2). Microscopically, the mass was found to be composed of mature fibroadipose tissue with islands of hematopoietic cells including scattered nucleated red blood cells and rare megakaryocytes. This was consistent with the diagnosis of a splenic myelolipoma. At 7 months of follow-up, the patient continued to do well.

## 3. Discussion

The authors present a rare symptomatic case of extra-adrenal myelolipoma in the spleen. Myelolipomas are nonfunctional, benign neoplasms composed of dense adipose and hematopoietic tissue. They are the second most common adrenal incidentaloma. Rarely, myelolipomas can present in extra-adrenal locations such as spleen, kidney, bones, or thorax [1]. The etiology of myelolipoma is unclear, but it is theorized that pathogenesis is a special case of extramedullary hematopoiesis that involves both mature fat tissue and clonal marrow elements. This suggests that the myeloid and lipomatous elements are from a common precursor [1]. With the wide distribution of extramedullary hematopoietic tissues through fetal development, it is feasible that myelolipomas of the spleen arise due to precursor cells that remain from development and contribute to local production of growth hormones or hematopoietic elements [1].

The majority of adrenal myelolipomas are found incidentally during the 6^th^ decade of life and followed with a surveillance protocol consisting of clinical monitoring for tumor progression [2]. If symptomatic, patients typically complain of vague abdominal pain thought to be due to mass effect from the tumor. Rarely, a patient may present with worsening flank pain due to hemorrhage [2]. CT is the first option for definitive diagnosis and usually reveals a well-circumscribed, round, hypodense, and heterogeneous mass. Fat density on imaging is one of the most useful factors in ascertaining a diagnosis of myelolipoma. Image-guided percutaneous biopsy can be employed to confirm diagnosis. Pathology reveals dense adipose tissue and trilinear hematopoietic cells. The recommended treatment of adrenal myelolipoma is conservative with surgery reserved for symptomatic patients.

As splenic myelolipomas are rare, no consensus exists as to work-up or treatment. Similar to previous case reports [3–6], our patient's myelolipoma was identified due to abdominal symptoms and was found on CT scan as a round, encapsulated splenic mass. While biopsy of adrenal fatty tumors is commonly employed for diagnosis, the risks of complications related to splenic biopsy are not insignificant. A recent study identified a minor complication rate of 7.2% (pain, minor bleeds) and a major complication rate of 1% (hemothorax requiring chest tube and blood transfusion) [7]. With this in mind, along with the enlarging mass, splenectomy was deemed to be the best option for both therapeutic and diagnostic purposes. Of note, splenectomy was the treatment of choice in the other reported splenic myelolipomas as well, due to similar symptoms and a concern for malignant nature of the mass. Like other reports, we also noted significant adhesions between the spleen and adjacent structures, likely secondary to inflammation from the tumor itself. Despite this, the patient had an uneventful operation and recovery.

In conclusion, this case underscores the importance of maintaining a wide differential diagnosis for splenic masses, including neoplastic and benign causes such as myelolipoma. In addition, while the prognosis of myelolipoma is excellent, surgery remains an appropriate treatment when concern for neoplasm is high.

## Data Availability

Data supporting the conclusions of the study are as outlined within the report. There is no need for further data for educational purposes, as well as secondary to HIPAA constraints.
